# Evaluation of APACHE II system among intensive care patients at a teaching hospital

**DOI:** 10.1590/S1516-31802003000200004

**Published:** 2003-03-05

**Authors:** Paulo Antonio Chiavone, Yvoty Alves dos Santos Sens

**Keywords:** APACHE II, Illness, Severity, Index, Outcome, Prediction, Prognosis, Intensive, Care, Unit, APACHE II, Índices, Prognósticos, Gravidade, Doença, Unidade Terapia, Intensiva

## Abstract

**CONTEXT:**

The high-complexity features of intensive care unit services and the clinical situation of patients themselves render correct prognosis fundamentally important not only for patients, their families and physicians, but also for hospital administrators, fund-providers and controllers. Prognostic indices have been developed for estimating hospital mortality rates for hospitalized patients, based on demographic, physiological and clinical data.

**OBJECTIVE:**

The APACHE II system was applied within an intensive care unit to evaluate its ability to predict patient outcome; to compare illness severity with outcomes for clinical and surgical patients; and to compare the recorded result with the predicted death rate.

**DESIGN:**

Diagnostic test.

**SETTING:**

Clinical and surgical intensive care unit in a tertiary-care teaching hospital.

**PARTICIPANTS:**

The study involved 521 consecutive patients admitted to the intensive care unit from July 1998 to June 1999.

**MAIN MEASUREMENTS:**

APACHE II score, in-hospital mortality, receiver operating characteristic curve, decision matrices and linear regression analysis.

**RESULTS:**

The patients’ mean age was 50 ± 19 years and the APACHE II score was 16.7 ± 7.3. There were 166 clinical patients (32%), 173 (33%) post-elective surgery patients (33%), and 182 post-emergency surgery patients (35%), thus producing statistically similar proportions. The APACHE II scores for clinical patients (18.5 ± 7.8) were similar to those for non-elective surgery patients (18.6 ± 6.5) and both were greater than for elective surgery patients (13.0 ± 6.3) (p < 0.05). The higher this score was, the higher the mortality rate was (p < 0.05). The predicted death rate was 25.6% and the recorded death rate was 35.5%. Through the use of receiver operating curve analysis, good discrimination was found (area under the curve = 0.80). From the 2 x 2 decision matrix, 72.2% of patients were correctly classified (sensitivity = 35.1%; specificity = 92.6%). Linear regression analysis was equivalent to r^2^ = 0.92.

**CONCLUSIONS:**

APACHE II was useful for stratifying these patients. The illness severity and death rate among clinical patients were higher than those recorded for surgical patients. Despite the stratification ability of the APACHE II system, it lacked accuracy in predicting death rates. The recorded death rate was higher than the predicted rate.

## RESUMO

**CONTEXTO:**

Pela complexidade do atendimento nas unidades de terapia intensiva e a própria situação dos pacientes, o prognóstico correto é de fundamental importância. Os índices prognósticos surgiram para auxiliar na avaliação do prognóstico destes pacientes.

**OBJETIVO:**

Avaliar o APACHE II (Acute Physiology and Cronic Health Evaluation II), analisando a estratificação dos pacientes quanto à letalidade, comparar a gravidade e condições de saída dos pacientes clínicos, pós-cirurgias eletivas, e pós-cirurgias de urgências; avaliar a capacidade do APACHE II de prever a letalidade hospitalar; e comparar a letalidade hospitalar observada com a esperada.

**TIPO DE ESTUDO:**

de acurácia, retrospectivo.

**LOCAL:**

Unidade de terapia intensiva da Santa Casa de Misericórdia de São Paulo (hospital terciário de ensino universitário).

**PARTICIPANTES:**

521 pacientes consecutivamente admitidos entre julho de 1998 a junho de 1999.

**VARIÁVEIS ESTUDADAS:**

Escore APACHE II, mortalidade intra-hospitalar. A acurácia do APACHE II foi avaliada pela curva *receiver operating characteristic*, pela decisão matricial e pela regressão linear.

**RESULTADOS:**

A idade média foi 50 ± 19 anos e a média do APACHE II foi de 16,7 ± 7,3. Entre os internados, 166 pacientes (32%) tinham doenças clínicas, 173 (33%) sofreram cirurgias eletivas e 182 (35%), cirurgias de urgências, sendo esta proporção estatisticamente similar. A média do APACHE II dos pacientes clínicos foi 18,5 ± 7,8, dos pacientes póscirurgias de urgências, 18,6 ± 6,5 e dos pacientes pós-cirurgias eletivas foi 13,0 ± 6,3. Quanto maior o APACHE II, maior a letalidade para todos os pacientes e para cada grupo. A letalidade prevista foi 25,6%, enquanto a observada foi 35,5%, com razão de mortalidade padronizada de 1,39. A análise da curva *receiver operating characteristic* mostrou boa discriminação do índice (área sob a curva = 0,80). Com a matriz de decisão 2 x 2, 72,2% dos pacientes foram corretamente classificados, sendo a sensibilidade 35,1% e a especificidade 92,6% (risco de 0,5). Quando a letalidade observada foi relacionada com a prevista, a análise de regressão linear mostrou r^2^ de 0,92.

**CONCLUSÕES:**

O APACHE II foi útil para estratificar os pacientes pela letalidade. A gravidade e a letalidade dos pacientes clínicos foram maiores que dos pacientes cirúrgicos. A capacidade do sistema APACHE II de estratificação foi boa, mas insuficiente para predizer com exatidão a letalidade destes pacientes. A letalidade observada foi maior que a prevista pelo APACHE II.

## INTRODUCTION

The high-complexity features of intensive care unit services and the clinical situation of patients themselves render correct prognosis fundamentally important not only for patients, their families and physicians, but also for hospital administrators, fund-providers and controllers. Prognostic indices have been developed for estimating hospital mortality rates for patients hospitalized in intensive care units, based on demographic, physiological and clinical data. The most frequently used indices are APACHE II (Acute Physiology and Chronic Health Evaluation II), APACHE III (Acute Physiology And Chronic Health Evaluation III), SAPS II (Simplified Acute Physiology Score II) and MPM II (Mortality Probability Model II).^1,4^

The APACHE II index consists of a score that takes account of the patient's age, chronic health condition and physiological variables (internal temperature, heart rate, respiratory rate, oxygenation, arterial pH, sodium, potassium, creatinine, hematocrit, white blood cells and Glasgow coma score). Although APACHE II was one of the first systems described, it is still the most widely used of them, insofar as the data required for its calculation are simple, well defined, reproducible, and collected on a routine basis during intensive care service provision. In Brazil, it is used by the Ministry of Health as a criterion for classifying intensive care units.^5^

Markgraf et al.^6^ compared the predictive capabilities of APACHE II, APACHE III and SAPS II and concluded that the three indices have good discriminating power and that APACHE II has the best calibration. For this reason, it scored the most accurate mortality prediction. Because of the differences between intensive care unit patients, we think it is necessary for every intensive care unit to have a prediction system which is validated for its specific kind of patients. Some factors for calculating mortality corrections must also be established, in order to help estimate the mortality of similar groups of patients in the same intensive care unit.

## OBJECTIVES

This study aimed to assess the APACHE II prognostic index in an intensive care unit at a Brazilian medical school hospital. The analysis of the capacity of the system for predicting hospital mortality rate was also an objective of this study, as well as to compare the hospital's recorded mortality rate with the expected mortality rate.

## METHODS

A total of 600 patients admitted to the 15 intensive care unit beds of Santa Casa de Misericórdia de São Paulo on a consecutive basis made up the initial assessment during the period from July 1998 to June 1999. This hospital is a tertiary and teaching facility of the Faculdade de Ciências Médicas da Santa Casa de São Paulo, Brazil. The study method was approved by the Institution's Research Ethics Committee.

For standardization purposes, 68 patients who remained for less than 24 hours in the intensive care unit were excluded from the study, as well as 11 others whose medical files could not provide all the required information. Patients were evaluated with regard to demographic information and hospitalization diagnosis, as well as their health condition at the time of discharge from hospital. They were divided into three groups, according to their kind of hospitalization: clinical patients, post-elective surgery patients and post-emergency surgery patients. Patients who came to the intensive care unit directly from the surgical theater or from the recovery room after post-anesthesia procedures were regarded as surgical.

For the APACHE II calculation, physiological variables were obtained within the first 24 hours of admission to the intensive care unit, as were the age and information on chronic disease. On the other hand, an equation established by Knaus et al. in 1985 was used for the calculation of mortality risk.^1^ In the case of sedated patients still under immediate post-anesthesia observation, the score relating to the assessment of consciousness level via the Glasgow Scale was calculated only after the patient had overcome the anesthetic effect. For intubated patients, this score was calculated considering their capacity to understand, regardless of speech. Recorded and expected mortality rates were compared for each group of patients, and the standard mortality ratio was calculated.

### Statistical analysis

Student's t test was used for comparing the averages of continuous measurements. The chi-squared test was used for comparing the proportions of categorized measurements and showing trends in situations of ranking. Averages across more than two groups were compared via analysis of variance between groups (ANOVA). The predictive capability of the index was assessed using the receiver operating characteristic curve, through a 2 x 2 decision matrix and linear regression analysis. The Statistical Package for the Social Sciences (SPSS) program, version 10.01, and *Epi Info* versions 6.04 and 2000 were used. p < 0.05 was considered statistically significant.

## RESULTS

The patients ranged from 13 to 91 years of age, with an average age of 50 ± 19 years. Three hundred and eighteen patients (61%) were male and 203 (39%) were female. With regard to the kind of hospitalization, 166 patients (32%) were clinical, 173 (33%) were post-elective surgery patients and 182 (35%) were post-emergency surgery patients, thus producing statistically similar proportions.

Among the clinical patients, the four most frequent diagnostic categories (67% of the total) were acute respiratory failure or insufficiency, sepsis, cardiac failure or insufficiency and post-cardiopulmonary arrest. The four most frequent post-elective surgery categories (75% of the total) were gastrointestinal surgery, post revascularization of the myocardium, heart valve surgery and transplantations (of liver and kidney). For the patients who underwent emergency surgery, the four most frequent categories recorded (70% of the total) were multiple trauma, abdominal surgery, surgery for sepsis or infection and head trauma. Trauma accounted for 15% of the diagnoses.

The patients’ average APACHE II score was 16.7 ± 7.3. The average APACHE II score for post-elective surgery patients was significantly lower than for the clinical patients and post-emergency surgery patients (respectively 13.0 ± 6.3, 18.5 ± 7.8 and 18.6 ± 6.5, p < 0.05). No differences between APACHE II averages for post-emergency surgery patients and clinical patients were recorded. Table 1 shows the distribution according to APACHE II score intervals and that 48% of the patients were in the interval between 11 and 20.

**Table 1:** Acute Physiology and Chronic Health Evaluation (APACHE II) scores of Intensive Care Unit patients at the Santa Casa de São Paulo Hospital, from July 1998 to June 1999

APACHE II Scores	Patients	
	N	%
0 – 5	23	4.4
6 – 10	94	18.0
11 – 15	116	22.3
16 – 20	134	25.7
21 – 25	90	17.3
26 – 30	41	7.9
> 30	23	4.4
**Total**	**521**	**100.0**

The average hospital mortality rate was 35.5%, with the highest mortality being that of clinical patients (53.6%), followed by post-emergency surgery patients (37.9%) and post-elective surgery patients (15.6%). The mortality rate recorded was higher than the predicted mortality (25.6%) by APACHE II, in that the mortality rate observed in clinical patients was substantially higher than expected, whereas for post-elective surgery patients and post-emergency surgery patients the mortality rates recorded were statistically similar to those expected. The standard mortality ratio for all patients was 1.39, ranging from 1.13 for post-emergency surgery patients to 1.43 for post-elective surgery patients and up to 1.67 for clinical patients (Table 2).

**Table 2:** Actual and expected hospital mortality rates via APACHE II and the standardized mortality rate (SMR) - Data compiled from 521 Brazilian intensive care unit patients’ medical files in the Intensive Care Unit of the Santa Casa de São Paulo Hospital, from July 1998 to June 1999

Patients	Actual mortality %	Expected mortality %	p	SMR
Non-surgical	53.6	32.1	0.0001	1.67
Elective surgery	15.6	10.9	0.2052	1.43
Emergency surgery	37.9	33.6	0.3815	1.13
**Total**	**35.5**	**25.6**	**0.0004**	**1.39**

*SMR = standardized mortality rate (actual/expected); χ^2^ comparing actual and expected mortality (significance was considered when p < 0.05).*

The comparison between APACHE II intervals and the mortality rate (Table 3) shows meaningful association between the APACHE II increases and the increase in mortality.

**Table 3:** APACHE II score ranges and deaths among 521 Brazilian intensive care unit patients of Santa Casa de São Paulo Hospital admitted from July 1998 to June 1999

APACHE II score ranges	Patients N	Deaths N	Deaths %
0 – 5	23	0	0
6 – 10	94	12	12.8
11 – 15	116	24	20.7
16 – 20	134	50	37.3
21 – 25	90	49	54.4
26 – 30	41	28	68.3
30	23	22	95.6
**Total**	**521**	**185**	**35.5**

*χ^2^ (linear trend) = 96.9; p < 0.001.*

The counting of patients who survived and those who died, for each level of death risk predicted, allowed the calculation of sensitivity, specificity and the percentage of correct predictions for each level of predicted death risk. Table 4 shows that the sensitivity of the calculated death risk was higher (90.3%) in level 0.1, and that it gradually decreased as the level increased, reaching 0% for level 1.0. Conversely, the specificity increased from 44.6% for level 0.1 up to 100% for death risk at 0.9. The most accurate prediction percentage (75.4%) occurred at death risk 0.4, with gradual decrease in accuracy when going upwards or downwards from this level. For death risk level 0.5, the correct classification was 72.2%, the sensitivity was 35.1% and the specificity was 92.6%.

**Table 4:** Sensitivity, specificity and correct classification according to levels of predicted death risk from 521 Brazilian intensive care unit patients of the Santa Casa de São Paulo Hospital admitted from July 1998 to June 1999

Predicted death risk	Sensitivity %	Specificity %	Correct classification %
0.1	90.3	44.6	60.8
0.2	75.7	69.9	72.0
0.3	60.5	82.7	74.8
0.4	49.2	89.9	75.4
0.5	35.1	92.6	72.2
0.6	23.8	96.7	70.8
0.7	13.5	98.8	68.5
0.8	7.0	99.4	66.6
0.9	2.2	100.0	65.3
1.0	0	100.0	64.5

**Figure 1:**
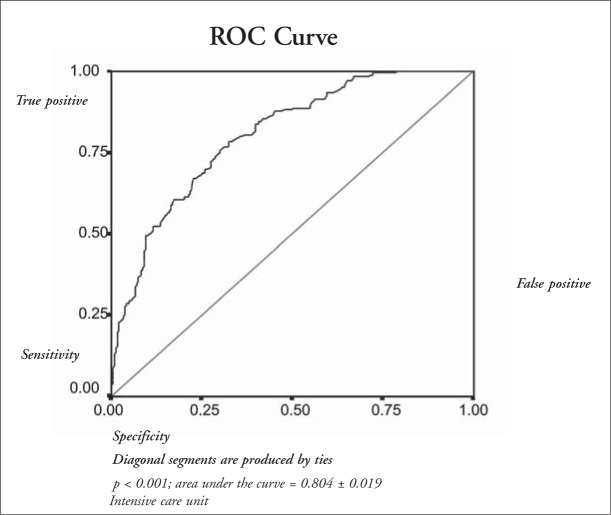
Receiver operating characteristic curve (ROC) from 521 Brazilian intensive care unit patients of the Santa Casa de São Paulo Hospital admitted from July 1998 to June 1999.

The receiver operating characteristic curve, based on the sensitivity and complemented specificity of predicted death risk, shows an area under the curve of 0.80 ± 0.02 (Figure 1).

**Figure 2:**
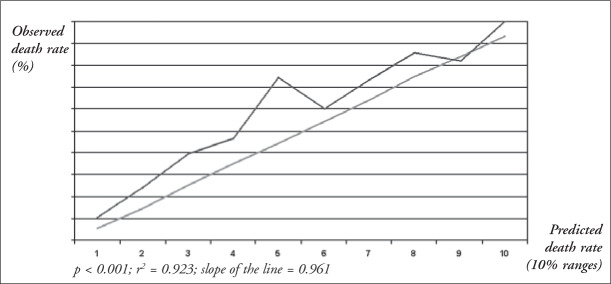
Calibration curve constructed by plotting observed death rate against predicted death rate, stratified into 10% risk ranges, from 521 Brazilian intensive care unit patients, of the Santa Casa de São Paulo Hospital admitted July 1998 to June 1999.

The calibration curve, with the death rates recorded as the ordinate and the rates of predicted death as the abscissa, stratified into 10% risk ranges, had a linear regression (r^2^) value = 0.923 (Figure 2).

## DISCUSSION

The analysis of the population studied showed that the three groups of patients (clinical, post-elective surgery and post-emergency surgery) had similar percentages and numbers of patients. This differed from the study of Knaus et al.,^7^ in which higher frequencies of clinical (47%) and post-elective surgery (40%) patients were recorded.

The percentage of post-emergency surgery patients that we recorded was higher than that recorded by most other studies.^6,7-11^ There was a higher frequency of trauma (15%) in relation to American and European studies, but this was similar to what has already been found in Brazil,^7,10,12^ The patient distribution in the APACHE II score intervals showed highest concentrations in the intermediate ranges, coinciding with what was found by other authors.^8,10,13^ However, the percentage of patients with APACHE II scores of less than 10 (22.4%), and thus with less severe illness conditions, was much lower than for the US study, 56%.^7^ The APACHE II average^14,15^ was higher than what was recorded in the United States,^7^ Europe^6,10^ and Japan,^8^ similar to what has been found in Brazil^16^ and Canada,^13^ and lower than what was recorded in Hong Kong.^14^

There was a meaningful connection between APACHE II scores and the mortality rate, for all the patients and for each diagnostic group. In each successive APACHE II score interval the mortality rate was higher than that of the preceding interval. Thus, this result has confirmed the capability of this index to stratify such patients according to the degree of severity of their health condition, as seen in the study by Knaus et al.^7^ and in Brazil.^11^

In conformity with the features mentioned, important differences can be observed between our study's patients and those of other studies that have assessed the applicability of APACHE II. The most relevant of these are: lower average age, higher percentage of post-emergency surgery patients, lower percentage of post-elective surgery patients, higher percentage of traumas, higher APACHE II average, and lower percentage of patients with APACHE II score lower than 10. Thus, our patients were younger, with a higher frequency of multiple trauma and acute surgical diseases that were more severe than those of the studies referred to.

On account of such differences, it has become important to assess the predictive capability of this prognostic index for particular patient populations. The ability of this gradational system to predict mortality rates for different patient groups has been assessed in several countries.^8,9,12-14,17-19^ The following items have been used for assessing the predictive capability of the APACHE II index: receiver operating characteristic curve, 2 x 2 decision matrix, and linear regression analysis (calibration curve).

Analysis of the receiver operating characteristic curve for hospital death prediction showed an area under the curve of 0.80, which was higher than the random prediction, but not enough to predict accurately the mortality rate of such patient populations. In comparing the capability of the APACHE II system for predicting mortality through this method, we observed that in this patient population it was meaningfully lower than what was recorded in the study by Knaus et al.^7^ and some from other countries,^9,13,14,19^ but similar to what was recorded in other studies,^6,8,17^ including one from Brazil.^20^

In the 2 x 2 decision matrix assessment, the most accurate classification percentage was obtained using criterion 0.4, which correctly classified 75.2% of the patients. With a 0.5 decision criterion, 72.2% patients were correctly classified. This correct classification was better than that recorded by random precision, yet not accurate enough to predict these patients’ mortality, and also substantially less accurate than the classification found in other countries for similar decision criteria.^1,6,14,17^ Thus, of the three statistical methods assessed, the APACHE II system showed good capability for stratifying this patient population according to mortality, with good discriminating power, good calibration, reasonable sensitivity and specificity, and a correct classification rate, but still with insufficient accuracy for predicting the mortality rate with precision.

The total mortality rate recorded was 35.5%. Mortality rates recorded in other countries have ranged from 16.9% to 36%^1,8,14,17^ and in Brazil from 38.1%^16^ to 40.5%.^15^ Costa et al.^11^ found a mortality rate of 28.5%, but their study considered intensive care unit mortality only, and not hospital mortality, as in other studies.

The predicted mortality rate was substantially lower than what was actually recorded, with a standard mortality ratio of 1.39, which was lower than the figure of 1.66 found in the multicenter study in Brazilian intensive care units.^12^ The standard mortality ratio has ranged from 0.59 to 1.58 in American hospitals, whereas in Europe it has ranged from 0.7 to 1.39.^7,17^ Bastos et al.^12^ assessed the use of APACHE II in a Brazilian multicenter study with 1.781 patients, and showed that it was able to stratify patients by mortality rate. However, a meaningful difference was found between the expected mortality rate and what was actually recorded, with a standard mortality ratio of 1.66 (variation from 0.95 to 2.4). Also in Brazil, Milani and Rocha^15^ found a standard mortality ratio of 1.20, but these authors only considered intensive care unit mortality, and not hospital mortality, as in this study and others mentioned earlier.

Many factors may explain the difference between the predicted mortality and what was actually recorded. These may include: the limitations of APACHE II, which is not a perfect prediction instrument; differences between this population and those of the studies that validated the index (some patient features like nutritional, ethnic, social, cultural and economic conditions); and its use in circumstances not applied by Knaus et al. (e.g. following revascularization of the myocardium).^7^ Other differences may include the criteria for selecting intensive care unit patients and also the availability of beds. Such aspects definitely affect the results, as well.

At the hospital where this study was carried out, the percentage of intensive care unit beds in relation to the total number is lower than in other countries, particularly the United States, where the APACHE II system was developed. Thus, at the time of the study by Knaus et al.,^7^ the percentage of intensive care unit beds in relation to the overall number of beds in United States’ hospitals was 5.6% and this increased to 10% by 1992.^18^ On the other hand, in Europe this percentage ranged from 2.6% to 3.8%, and in Japan it was 2%.^8^ At our hospital, it was 2.5%, thus demonstrating the limited availability of intensive care unit beds. The characteristics of such a tertiary hospital school, which is a referral center for multiple trauma patients and highly complex procedures, underline the need for a larger number of intensive care unit beds. Some other relevant differences with the other countries involved, which have an influence on the results, relate to health and cost policies, as well as financial conditions and resources made available.

Although the APACHE II index was not developed for assessing individual prognoses, intensive care unit physicians and medicine as a whole have yearned for such predictive ability. Thus, many studies have attempted to assess the use of this index with this purpose in mind.^13^ In this study, an absence of specificity for predicting individual death was noticed. Therefore, for individual procedures, we cannot depend only on this index and its formula for calculating death risk. Other issues that underlie these decisions, including those of an ethical nature, must be respected.^21^ However, APACHE II may be useful as an additional instrument for backing up clinical decisions.

This study showed that in this population, APACHE II was capable of stratifying patients according to illness severity in relation to mortality. However, it was not as accurate as in other studies.^7,8,13,14,19^ It had good discriminating power for distinguishing patients who survived from those who died; it also had good calibration, but it was generally not sensitive, specific and accurate enough to predict the patients’ exact mortality. Likewise, it was not accurate enough to predict the patients’ individual mortality. Thus, on account of the differences amongst intensive care unit patients, each intensive care unit needs to have a prediction system that is validated for its specific kind of patients, and needs to verify its standard mortality rate. Some factors for calculating mortality corrections must also be established, in order to help estimate the mortality of similar patient groups in the same intensive care unit. It is just as important to develop and perfect indices that assess not only the patients’ mortality rate, but also their quality of life after hospitalization within intensive care units. In our intensive care unit, we have been using this prognosis index and attempting to determine correction factors so as to improve the capacity for estimating patient prognoses.

## CONCLUSION

The APACHE II prognostic index was useful for stratifying patients according to the severity of their health condition. The higher the APACHE II score was, the higher the mortality rate was. The severity and mortality rates were different and they were assessed in the following decreasing order: clinical patients, post-emergency surgery patients and post-elective surgery patients. The predictive capability of APACHE II was good, but not enough to accurately predict the mortality among the population studied. The recorded mortality rate was similar to the predicted rate for surgical patients and higher than what was predicted for clinical patients. In general terms, the recorded mortality rate was higher than expected from the APACHE II score.
